# Phone calls to enhance PrEP persistence among Kenyan women accessing postabortal care: a cluster randomized trial

**DOI:** 10.3389/frph.2025.1709721

**Published:** 2025-11-27

**Authors:** Renee Heffron, Lydia Etyang, Bernard Nyerere, Inviolata Wanyama, Yasaman Zia, Torin Schaafsma, Katherine K. Thomas, Margaret Mwangi, Lavender June, Felix O. Mogaka, Catherine Kiptinness, Michael Kamiru, Taryn Barker, Hideaki Okochi, Monica Gandhi, Kenneth Ngure, Elizabeth A. Bukusi, Nelly R. Mugo

**Affiliations:** 1 Department of Medicine, University of Alabama at Birmingham, Birmingham, AL, United States; 2 Department of Global Health, University of Washington, Seattle, WA, United States; 3 Partners in Health and Research Development, Kenya Medical Research Institute, Nairobi, Kenya; 4 Center for Microbiology Research, Kenya Medical Research Institute, Nairobi, Kenya; 5 Marie Stopes Kenya, Nairobi, Kenya; 6 Department of Obstetrics, Gynecology and Reproductive Sciences, University of California San Francisco, San Francisco, CA, United States; 7 Children’s Investment Fund Foundation, Nairobi, Kenya; 8 Children’s Investment Fund Foundation, London, United Kingdom; 9 Department of Medicine, University of California San Francisco, San Francisco, CA, United States; 10 School of Public Health, Jomo Kenyatta University of Agriculture and Technology, Nairobi, Kenya; 11 Department of Obstetrics & Gynecology, University of Washington, Seattle, WA, United States; 12 Center for Clinical Research, Kenya Medical Research Institute, Nairobi, Kenya

**Keywords:** HIV prevention, pre-exposure prophylaxis, postabortal care, Kenya, young women

## Abstract

**Introduction:**

In Kenya, young women face dual epidemics of HIV and unintended pregnancy, yet provision of HIV pre-exposure prophylaxis (PrEP) in reproductive health settings is uncommon. We aimed to estimate PrEP uptake and persistence when PrEP was integrated into services for people seeking postabortion care (PAC) and to determine whether enhancing the PrEP program with an adherence intervention—simple phone calls—impacted PrEP persistence and use.

**Methods:**

PAC clinics in Kenya launched PrEP delivery and were randomized to conduct enhanced support (weekly calls during Month 1, biweekly in Month 2, and monthly thereafter) or standard of care (SOC) for PrEP adherence and retention. The primary outcome for the cluster randomized trial was PrEP refills at one month. PrEP refills and adherence [collected on a subset through point-of-care urine tenofovir (TFV) testing] were compared among participants accessing PrEP at facilities assigned to offer enhanced vs. SOC support via Poisson regression models.

**Results:**

From April 2021 to March 2023, 8,362 women sought PAC from participating facilities. Fifty-five percent of women received PrEP information, 73% of those had HIV testing, and 36% of those received counseling and initiated PrEP. After the trial launch, 4,112 women sought PAC, and 655 (15.9%) initiated PrEP. At Month 1, 63/408 (15.4%) women in facilities who were randomized to the enhanced arm and 14/247 (5.7%) in the SOC arm received a PrEP refill [relative risk (RR) = 2.7, 95% CI: 0.90–8.2]. TFV was detected at Month 1 in 19.0% of the enhanced arm and 9.4% of the SOC arm (RR = 2.03, 95% CI: 0.89–4.65).

**Conclusions:**

We observed large gaps in the provision of PrEP information and PrEP counseling that contributed to low PrEP uptake in PAC clinics. Among women who initiated PrEP, persistence and adherence were low. Phone calls yielded a statistically significantly higher retention at Month 1, a finding that may warrant further investigation.

## Introduction

In Kenya, young women experience a tripartite of parallel sexual and reproductive health epidemics with HIV, unintended pregnancy, and sexual violence (1). Unintended pregnancy is common in the region, with nearly half being unplanned, mistimed, or undesired (2). Induced abortion is illegal in Kenya except in cases where there is a need for emergency treatment or when the life or health of the mother is in danger (3). However, young women often experience abortion, with rates highest among those aged 20–24 years (4). Women experiencing spontaneous or induced abortion can seek care from a postabortion clinic that provides emergency obstetric care, medical stabilization, treatment for complications, and counseling (5). While this service is meant to be one-time with a modest amount of follow-up after discharge, it offers an opportunity to explore whether additional sexual and reproductive health services, including HIV prevention services, could be integrated.

Daily oral TDF-based medication is the most common PrEP medication available in Kenya, and to date, more than 500,000 people have initiated PrEP (6). Daily oral PrEP is a user-controlled HIV prevention strategy that overcomes many of the challenges associated with condoms, male circumcision, and reliance on partners with HIV to maintain viral suppression. For women, access to PrEP has been facilitated by making it available at family planning and perinatal clinics, which are commonly utilized by young women (7–9). To our knowledge, PrEP has never been integrated into postabortion care (PAC), which is another type of reproductive healthcare service that could capitalize on the routine sexual health counseling already provided and include discussion and offering of PrEP. In a prior study among women seeking postabortion care, we found a chlamydia prevalence of 18% and a high frequency of HIV risk indicators, including condomless sex, new partnerships, and unknown HIV status of partners (10). Women in this study expressed a strong interest in HIV pre-exposure prophylaxis (PrEP), which is approved for use during pregnancy and lactation. However, PrEP access required linkage to a different clinic or facility, and actual use remained low at 2.5%.

For the current study, we aimed to estimate PrEP uptake and persistence when PrEP was integrated into services for people seeking postabortion care. For young women who initiate daily oral PrEP, persistence and adherence to the daily pill regimen are often challenged by individual, interpersonal, social, and structural factors (11). Among these barriers, stigma is extremely challenging to combat (12). Programs tailored to the preferences and lifestyles of young people and offering adherence interventions have seen better adherence and persistence (11). Thus, for our study, we incorporated a design facilitating the determination of whether enhancing the PrEP program with an adherence intervention—simple phone calls—impacted PrEP persistence and use.

## Methods

### Study design, setting, and participants

This study, conducted from April 2021 to April 2023 in Nairobi, Murang'a, Kiambu, and Kisumu counties of Kenya, involved public and private clinics providing postabortion services and included two phases. Clinics were selected for inclusion based on recommendations from partner leadership [e.g., Marie Stopes Kenya (MSK), county government health leadership in HIV and reproductive health], the volume of postabortion clients, and the willingness of the clinic leadership to participate. Halfway through the study, one clinic was failing to provide PrEP services to clients, and it was replaced by a 15th clinic. Throughout the study, three technical assistance teams oversaw research operations at each facility: Kenya Medical Research Institute (KEMRI) research teams from Thika (the KEMRI Partners in Health and Research Development group) and Kisumu (the KEMRI Research on Care, Treatment, and Prevention group), and MSK. Since MSK Kenya had less experience implementing PrEP research trials, the KEMRI research staff also worked with the MSK teams as needed to envision the program and support its operations with PrEP.

#### Programmatic foundation

In Phase 1, PrEP delivery was launched in 14 facilities at the point of PAC service delivery for the first time in these clinics. PrEP launch entailed provider training about PrEP clinical and operational delivery, linking the clinic to an existing PrEP supply at a Ministry of Health-supported facility, and establishing continuous technical assistance to each facility. Data from <30-years-old women were abstracted from patient medical charts for demographics, HIV testing, and PrEP prescribing, which also began during this phase (13). The first day of PrEP delivery in each postabortion clinic was different, ranging from April to December 2021. All major operations were conducted by existing clinic staff, and the research staff were available to provide technical support.

#### Cluster randomized trial

Phase 2 began once all clinics were delivering PrEP, and those assigned to the enhanced support (intervention) arm began conducting the phone call intervention. Randomization was stratified by clinic volume and public/private designation, and clinics were assigned 1:1 to the standard of care (SOC) or intervention (enhanced support) arm. Subsequently, clinics randomized to the standard of care arm continued to implement PrEP delivery without any change. In April 2022, clinics randomized to the intervention arm began enhanced PrEP delivery by adding routine phone calls to PrEP clients after they initiated PrEP. Based on experience and literature with antiretroviral initiation (14, 15), calls were made on Days 4, 10, and 28 (with an additional call on Day 32 if needed) within the first month after initiation, tapering to every 2 weeks during the subsequent month and once every month thereafter. Conversation during the calls, which were conducted by KEMRI research staff and MSK call center staff, followed a basic script that encouraged open dialogue about how the patient was doing with PrEP, inviting discussion about challenges and discomforts related to the medication, and providing reminders of upcoming appointments. For each challenge/discomfort mentioned, the staff member would probe for details and discuss possible solutions to overcome the challenge. For study purposes, the date at which all enhanced facility clinics began phone calls was the date used to begin counting time in the trial phase.

#### Nested research components for a subset of program clients

During the trial, a subset of PrEP clients from both arms was offered enrollment into a research cohort with quarterly longitudinal follow-up for 6 months. These procedures included completing interviewer-administered questionnaires to capture self-reported demographics, HIV testing, PrEP adherence, sexual behavior, and symptoms of vaginal infections. Participants were asked to provide a urine sample that was used with a validated point-of-care lateral flow qualitative assay to determine the presence of tenofovir (TFV), a marker of oral TFV use within the past week (16–18). The results from this testing were returned to participants and incorporated into PrEP counseling.

#### Data collection and management

Data were abstracted from clinic records, particularly the standard form in Kenyan government facilities known as the “PrEP Clinical Encounter Form” and through case report forms for research data. All data were directly entered into a web-based data management system, REDCap (Institute for Translational Health Sciences (ITHS) University of Washington) (19, 20). To capture offers of PrEP and enable linkage to the clinic PrEP log, we created a simple form for each client file (a “PrEP offer” form) that was completed by clinic staff when a client was discharged.

#### Statistical methods

Our primary outcome for this cluster randomized trial (CRT) was PrEP refill 1 month (20–44 days) after PrEP initiation. We used modified Poisson regression models (21) with generalized estimating equations robust standard errors and an independent working correlation structure to estimate relative risks comparing the frequency of having a PrEP refill at 1 month among clinics assigned to the enhanced support vs. standard of care arms. Using the same statistical method, we also compared the frequency of additional outcomes among intervention vs. standard of care clinics: PrEP refill 3 months (45–104 days) after initiation and PrEP refill 6 months (105–266 days) after initiation, and ever receiving a PrEP refill during the study period. To estimate differences in PrEP adherence by arm among women in the research cohort, we used separate models to compare the frequency of (1) self-reported “good” adherence (as opposed to “bad” or “fair”) and (2) urine TFV positivity (i.e., detected). In sensitivity analyses of adherence (assessed via self-report and the urine TFV test), we assumed that all women missing data were non-adherent (e.g., were not engaged with the study or PrEP use).

## Results

### Clinic and participant characteristics

Of the 15 engaged clinics, 6 were public facilities and 9 were private (Supplementary Table 1). Of the private facilities, 6 were managed by MSK, and the others were independently owned. Two of the MSK clinics had a history of having very high volumes of clients, driving their overall volume higher than any of the others.

**Figure 1:**
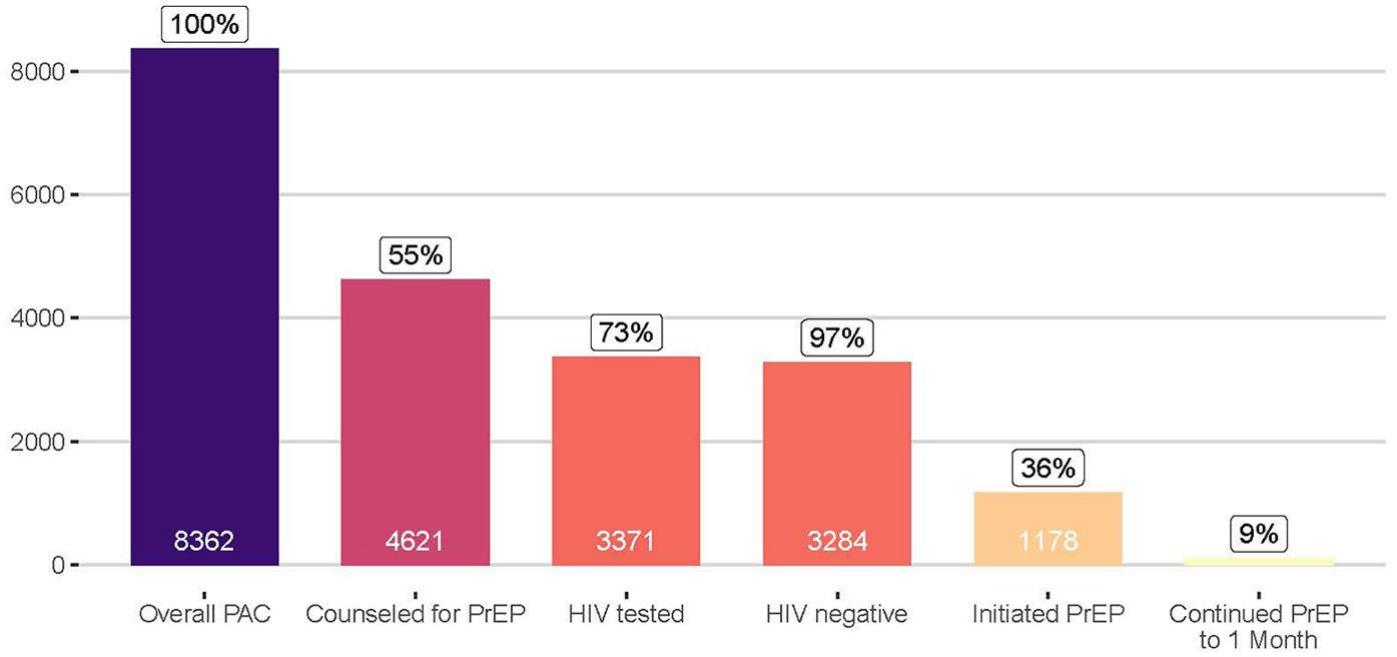
Continuum of PrEP counseling, HIV testing, and PrEP use among women accessing postabortion services at participating clinics in Kenya.

During the 2-year study period, engaged postabortion clinics received 8,362 clients. Clients had a median age of 24 years [interquartile range (IQR): 22–27], 53% were married or cohabiting, and 19% were currently attending school (Supplementary Table 2). Clients accessing services at Marie Stopes-supported clinics were different from others, in that they were predominantly never married (86%), and 39% were currently attending school. Of the 8,362 clients, 55% were counseled about PrEP, of whom 73% were tested for HIV, 97% of those tested were HIV negative, and 36% of HIV-negative clients initiated PrEP (Figure 1). Of the 1,178 initiating PrEP, 9% returned for a refill 1 month later, and 13% returned at any point to a postabortion clinic engaged for this program. Data describing reasons that women declined PrEP were available from 3,429 women (99% of those counseled about PrEP who declined), and their top reasons included being in a stable partnership (cited by 56%), needing more time to think about PrEP (16%), having plans to abstain from sex (11%), and concerns about the pill burden (9%), potential side effects (5%), and potential judgement from others (3%) (Supplementary Figure 1).

### Nested CRT outcomes—trial numbers and consort diagram

**Figure 2:**
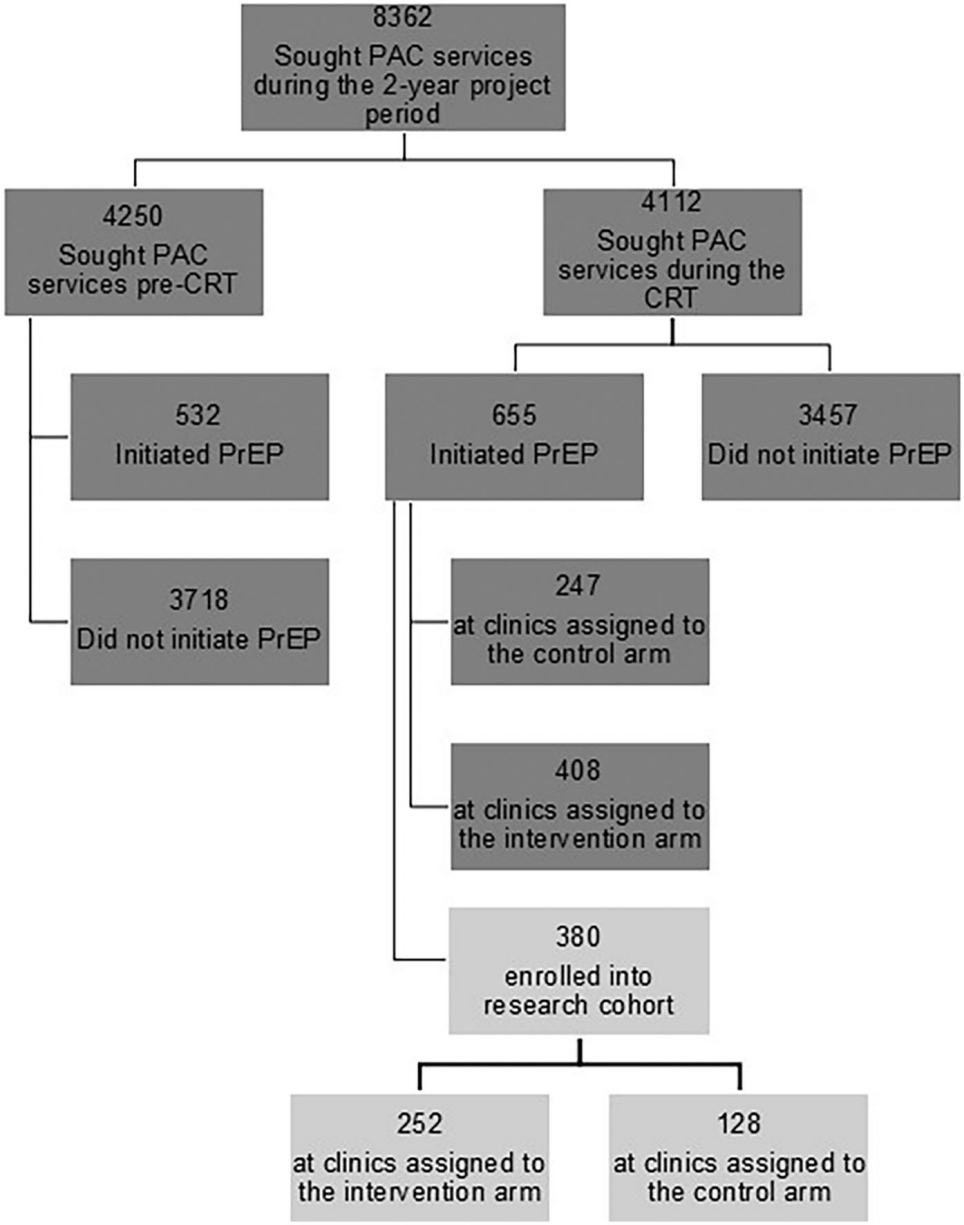
CONSORT diagram of women seeking postabortion care and included in data captured before and during cluster randomized trial execution. PAC, postabortion care; CRT, cluster randomized trial; PrEP, pre-exposure prophylaxis.

Subsequent to randomization and intervention launch, 655/4,112 (15.9%) women seeking services at study postabortion clinics initiated PrEP through engaged postabortion clinics (*N* = 408 through clinics assigned to the intervention arm and 247 through clinics in the standard of care arm, Figure 2). While the distribution of women was similar by clinic type and volume, clinics supported by the KEMRI Thika team contributed substantially more to the intervention arm clinics than standard of care clinics (Table 1). Demographics characteristics of women in standard of care and intervention clinics were similar except that women at intervention clinics had greater frequency of having a partner with “high risk and unknown HIV status” (87 vs. 65%) and inconsistent/no condom use (58 vs. 33%) as per the standard form in Kenyan government facilities known as the “PrEP Clinical Encounter Form” (Table 2).

**Table 1:** Clinic characteristics by randomized arm.

Clinic characteristics		Total (*n* = 14)		Standard of care (*n* = 7)		Intervention (*n* = 7)	
		*N*	(%)	*N*	(%)	*N*	(%)
Research technical assistance partner	KEMRI Thika	4	(29%)	1	(14%)	3	(43%)
	KEMRI Kisumu	4	(29%)	2	(29%)	2	(29%)
	Marie Stopes Kenya	6	(43%)	4	(57%)	2	(29%)
Clinic type	Public	6	(43%)	3	(43%)	3	(43%)
	Private	8	(57%)	4	(57%)	4	(57%)
Clinic volume	High	6	(43%)	3	(43%)	3	(43%)
	Low	8	(57%)	4	(57%)	4	(57%)

KEMRI, Kenya Medical Research Institute.

**Table 2:** Baseline characteristics of cluster randomized trial cohort by randomization arm.

Participant characteristics	Total (*n* = 655)		Standard of care (*n* = 247)		Intervention (*n* = 408)	
	*n*/*N*	(%)	*n*/*N*	(%)	*n*/*N*	(%)
Implementing partner						
KEMRI Thika	238	(36%)	1	(<1%)	237	(58%)
KEMRI Kisumu	157	(24%)	145	(59%)	12	(3%)
Marie Stopes Kenya	260	(40%)	101	(41%)	159	(39%)
Clinic type						
Public	383	(58%)	146	(59%)	237	(58%)
Private	272	(42%)	101	(41%)	171	(42%)
Clinic volume						
High	500	(76%)	155	(63%)	345	(85%)
Low	155	(24%)	92	(37%)	63	(15%)
Age, median (IQR)	22	(20–25)	22	(20–25)	22	(20–25)
Marital status						
Cohabitating/married	190/603	(32%)	55/221	(25%)	135/382	(35%)
Never married	394/603	(65%)	161/221	(73%)	233/382	(61%)
Divorced	19/603	(3%)	5/221	(2%)	14/382	(4%)
Widowed	0/603	(0%)	0/221	(0%)	0/382	(0%)
Currently attending school	108/466	(23%)	49/168	(29%)	59/298	(20%)
Sex partner living with HIV	0	(0%)	0	(0%)	0	(0%)
Sex partner high HIV risk, unknown status	514	(78%)	160	(65%)	354	(87%)
Multiple sex partners	79	(12%)	31	(13%)	48	(12%)
Ongoing experience with intimate partners violence/gender-based violence	7	(1%)	4	(2%)	3	(<1%)
Transactional sex	14	(2%)	6	(2%)	8	(2%)
STI in prior 6 months	37	(6%)	19	(8%)	18	(4%)
Recurrent use of PEP	12	(2%)	3	(1%)	9	(2%)
Recurrent sex with alcohol/recreational drugs	11	(2%)	1	(<1%)	10	(2%)
Inconsistent or no condom use	316	(48%)	81	(33%)	235	(58%)
Injection drug use with shared needles	0	(0%)	0	(0%)	0	(0%)
Signs/symptoms of STI	2/639	(<1%)	0/239	(0%)	2/400	(<1%)
Tested negative for HIV	653	(>99%)	247	(100%)	406	(>99%)
In research cohort	380	(58%)	128	(52%)	252	(62%)

KEMRI, Kenya Medical Research Institute; IQR: interquartile range; STI, sexually transmitted infection; PEP, post exposure prophylaxis.

Of PrEP initiators during the cluster randomized trial phase, 380 (58%) enrolled into the research cohort (*N* = 252 in intervention clinics and *N* = 128 in standard of care clinics). Clinic characteristics, age, marital status, and school attendance were distributed similarly among PrEP-initiating women in the pre-CRT and CRT phases (Supplementary Table 3). While demographics of the research cohort were similar to those not enrolled in research, there were differences in the clinic characteristics, with a higher proportion of the research cohort being from KEMRI-supported, public, and high-volume clinics (Supplementary Table 4).

### Nested cluster randomized trial outcomes—intervention fidelity and trial outcomes

Of all participants eligible to receive intervention phone calls, 82% of calls were attempted, of which 90% were successful. Month 1 retention was higher at clinics in the intervention arm (25%) than the standard of care arm (8%), and this was a statistically significant difference [relative risk (RR) 3.25, 95% confidence interval (CI): 1.05–10.09, Table 3]. Our primary outcome, receiving a PrEP refill 1 month after initiation, was also greater in the intervention arm (15% vs. 6% in the standard of care arm, Supplementary Figure 2) but did not reach statistical significance (RR = 2.72, 95% CI: 0.90–8.23, Table 3). Other refill-related outcomes also trended this way: PrEP refill 3 months after initiation (RR = 1.82, 95% CI: 0.42–7.92), PrEP refill 6 months after initiation (RR = 6.66, 95% CI: 1.09–40.6), ever receiving a PrEP refill (RR = 2.52, 95% CI: 0.80–7.93). Month 1 adherence to PrEP was measured in the research cohort, and 76% of those attending the clinic self-reported high adherence (74% intervention arm, 86% standard of care arm, RR = 0.86, 95% CI: 0.7–1.05). Of 113 women administered the point-of-care urine test for TFV, 53% were positive for TFV presence (51% in intervention clinics vs. 63% in standard of care clinics, RR = 0.81, 95% CI: 0.54–1.20). When extrapolating to all research cohort women and assuming that the lack of self-reported adherence/urine test equates to non-adherence, 23% of women in the intervention clinics were adherent vs. 9% in the standard of care clinics (RR = 2.50, 95% CI: 1.33–4.70), and 19% of women in the intervention clinics had TFV positivity vs. 9% in standard of care clinics (RR = 2.03, 95% CI: 0.89–4.65).

**Table 3:** Estimated effect of enhanced adherence package on PrEP use and adherence (CRT cohort).

Retention, continuation, or adherence metric	Total		Standard of care		Intervention		Intervention vs. standard of care		
	*n*/*N*	(%)	*n*/*N*	(%)	*n*/*N*	(%)	RR	(95% CI)	*p*-value
Program retention in the program cohort (*N* = 655 total, 247 standard of care and 408 intervention)									
Retained at M1	121/655	(18%)	19/247	(8%)	102/408	(25%)	3.25	(1.05–10.09)	0.041
PrEP continuation in the program cohort (*N* = 655 total, 247 standard of care and 408 intervention)									
At M1 (20–44 days from initiation) [PRIMARY]	77/655	(12%)	14/247	(6%)	63/408	(15%)	2.72	(0.90–8.23)	0.076
At M3 (45–104 days from initiation)	32/655	(5%)	8/247	(3%)	24/408	(6%)	1.82	(0.42–7.92)	0.43
At M6 (105–266 days from initiation)	12/655	(2%)	1/247	(<1%)	11/408	(3%)	6.66	(1.09–40.60)	0.040
Ever refilled	98/655	(15%)	19/247	(8%)	79/408	(19%)	2.52	(0.80–7.93)	0.12
PrEP adherence in the research cohort (*N* = 380 total, 128 standard of care and 252 intervention)									
Adherent at M1 (self-report)	71/94	(76%)	12/14	(86%)	59/80	(74%)	0.86	(0.70–1.05)	0.15
Adherent at M1 (self-report, missing = assumed non-adherent)	71/380	(19%)	12/128	(9%)	59/252	(23%)	2.50	(1.33–4.70)	0.005
Adherent at M1 (urine test)	60/113	(53%)	12/19	(63%)	48/94	(51%)	0.81	(0.54–1.20)	0.30
Adherent at M1 (urine test, missing = assumed non-adherent, research cohort only)	60/380	(16%)	12/128	(9%)	48/252	(19%)	2.03	(0.89–4.65)	0.094

M, month.

## Discussion

To our knowledge, this is the first report of a PrEP program taking place in clinics designed to provide postabortion care in Kenya. PrEP uptake was quite low in the study—14% of the >8,000 women overall and 36% of those who received an HIV-negative test result. However, it's important to note that >3,300 HIV tests were conducted in clinics where a majority had not previously done HIV testing, and >1,100 women initiated PrEP through this means. In fact, the facilities achieved HIV testing among 73% of the people who were determined to need HIV and PrEP counseling, which was approximately 40% of all women seeking postabortion services. This frequency is higher than expected since HIV testing was not being routinely done before our study and may reflect an impact of the PrEP program. Refills through the postabortion clinics 1 month later were also uncommon—12% overall. The low number of refills may not be surprising, given the nature of these clinics to provide a service that is meant to be one-time and the numerous sources of stigma associated with being at a postabortion clinic.

Our phone call intervention was feasible to implement, and it may have increased the frequency of refills and pill taking—but we didn't have enough PrEP use to know whether this difference was statistically significant. In Kenya, programs to support care and retention of people with HIV have long used telephone calls and messaging to reach out to people frequently to build rapport with the facility, staff, and program. Thus, it is not surprising that a parallel approach for PrEP would also be successful.

Thus far in oral PrEP programs, persistence in accessing refills and adherence to the daily oral medication have been the “Achilles heels.” We used an objective measure of adherence (point-of-care urine testing for tenofovir) that has been shown to yield more accurate counseling sessions (22) and support adherence in some settings (23, 24). But this testing platform is limited to detecting tenofovir that was ingested within the past 7 days and is not a measure of consistent use during the past month. Other diagnostic tools are available to determine longer-term PrEP adherence, but they require more complicated sample collection (e.g., blood or hair) and specialized laboratory techniques that are not available in Kenya. For this science-driven study, the use of the point-of-care assay held numerous advantages.

In the future, novel PrEP products, such as the monthly dapivirine ring or 6-monthly lenacapavir injections, may become available in Kenya and could be considered to be integrated into postabortion care settings. These products are proposed to offer fewer challenges to PrEP adherence and offer very discrete options.

Our study had numerous limitations. The program data are simple and leave numerous questions about program drop-out unaddressed. Existing tools for monitoring and evaluation are limited to tracking people who seek services at the clinic and are not able to be linked to external services (e.g., family planning provided at another facility). Additionally, we had to create a “PrEP offer” form to capture PrEP initiation and refills from the postabortion provider, and this was new to providers, which created challenges to obtaining high-quality data. Even with this data collection strategy, we may have missed data on PrEP refills or other HIV prevention services if people sought them from other locations, and this would have impacted our primary analysis of program retention. We assume that missing data on PrEP refills were evenly distributed between intervention and control clinics, and thus our results are likely biased toward the null. In addition, our statistical models are not adjusted for possible confounding due to imbalances we observed in individual characteristics (e.g., sexual behavior). Thus, our results should be interpreted as overall effects, rather than being independent of the behaviors and characteristics of our participants. Finally, embedding research elements into the trial yielded a research cohort with greater engagement than those who declined research participation and may have contributed to our PrEP refill rates at Month 1. In our analysis of adherence, we conducted a conservative sensitivity analysis where we assumed that missing data came from non-adherent participants, and thus, the frequency of high adherence is very low in this analysis.

## Conclusions

In summary, integrating PrEP into postabortion services facilitated reaching young women with potential exposure to HIV who may not otherwise be reached. This population experiences multiple sources of stigma that create important vulnerabilities and should not be overlooked. Postabortion clinics offer a novel space to consider delivering PrEP, and women managing the recent loss of pregnancy need extra support to be able to transition from thinking about their very immediate reproductive health needs to HIV prevention in the near and longer term. We observed low persistence with PrEP refills at the PAC itself. To further consider postabortion clinics as a setting to initiate PrEP, additional linkage to family planning or other similar settings may facilitate better access to PrEP refills and persistence. Additionally, the integration process itself needs to recognize the importance of providing foundational PrEP knowledge and the important role of continuous technical support, as providers in these spaces are not accustomed to providing PrEP or counseling on PrEP. Finally, as novel prevention methods become available in Kenya, their integration into different types of reproductive health spaces can capitalize on the experiences with oral PrEP to yield fruitful opportunities for some of the people most vulnerable to HIV.

## Data Availability

Individual participant data that underlie the results reported in this article, after de-identification, can be shared via request and after consideration of the study primary investigators. Send email to the corresponding author to initiate discussion. The study protocol and statistical analysis plan are readily available by emailing the corresponding author.
