# Supplementary figures and images for: Phone calls to enhance PrEP persistence among Kenyan women accessing postabortal care: a cluster randomized trial
Source: Front Reprod Health. 2025 Nov 27;7:1709721. doi: 10.3389/frph.2025.1709721 (PMC12695733; doi:10.3389/frph.2025.1709721)

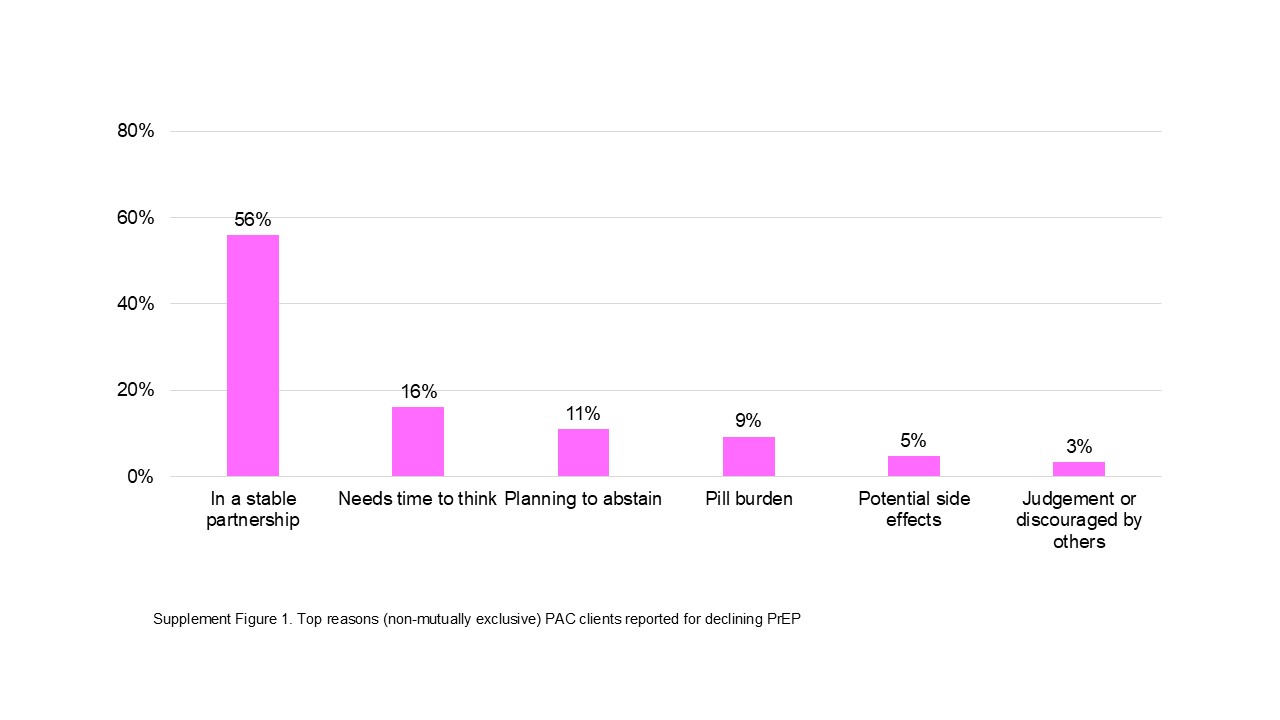

Supplement: Supplementary file 1 [file Image1.jpeg]

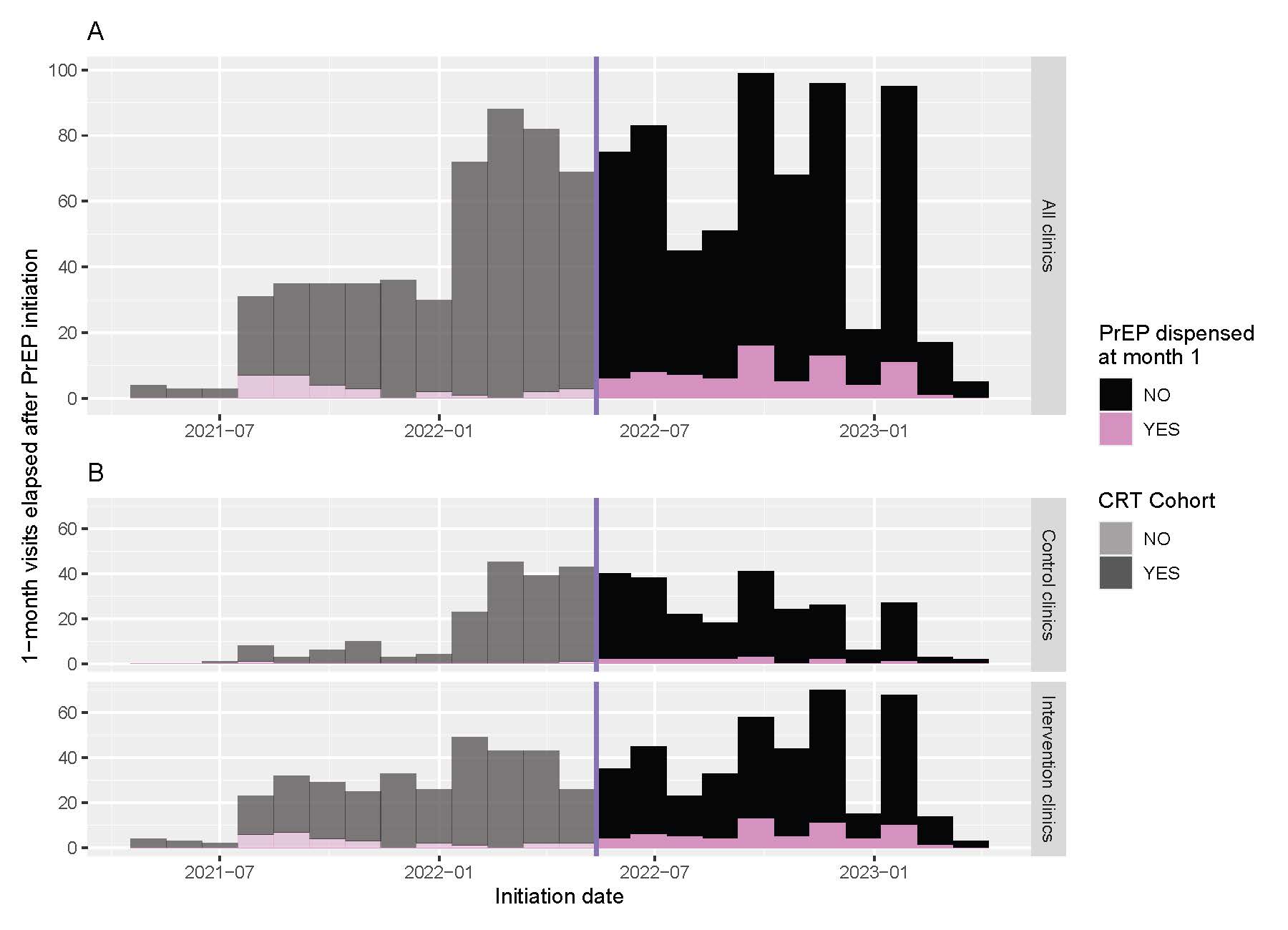

Supplement: Supplementary file 2 [file Image2.jpeg]
