# Supplementary material for: Phone calls to enhance PrEP persistence among Kenyan women accessing postabortal care: a cluster randomized trial
Source: Front Reprod Health. 2025 Nov 27;7:1709721. doi: 10.3389/frph.2025.1709721 (PMC12695733; doi:10.3389/frph.2025.1709721)
Supplement: Supplementary file 3 [file Table1.docx]

**Supplementary table 1: Facility characteristics by research technical assistance partner**

|  |  | **Total (n=15)**  **N (%) or Median (IQR)** | | **KEMRI Thika (n=4)**  **N (%) or Median (IQR)** | | **KEMRI Kisumu (n=5)**  **N (%) or Median (IQR)** | | **Marie Stopes Kenya (n=6)**  **N (%) or Median (IQR)** | |
| --- | --- | --- | --- | --- | --- | --- | --- | --- | --- |
| Number of postabortion care clients |  | 589 | (224–796) | 560 | (238–892) | 89 | (66–291) | 700 | (652–1079) |
| Clinic type | Public | 6 | (40%) | 4 | (100%) | 2 | (40%) | 0 | (0%) |
|  | Private | 9 | (60%) | 0 | (0%) | 3 | (60%) | 6 | (100%) |
| Clinic volume | High | 6 | (40%) | 2 | (50%) | 2 | (40%) | 2 | (33%) |
|  | Low | 9 | (60%) | 2 | (50%) | 3 | (60%) | 4 | (67%) |

**Supplementary table 2: Baseline characteristics for observational (programmatic) cohort by research technical assistance partner**

|  | **Total (n=8362)**  **n/N (%) or median (IQR)** | | **KEMRI Thika**  **(n=2278)**  **n/N (%) or median (IQR)** | | **KEMRI Kisumu**  **(n=1096)**  **n/N (%) or median (IQR)** | | **Marie Stopes Kenya**  **(n=4988)**  **n/N (%) or median (IQR)** | |
| --- | --- | --- | --- | --- | --- | --- | --- | --- |
| Age, Median (IQR) | 24 | (22–27) | 24 | (21–27) | 24 | (21–27) | 25 | (22–27) |
| Marital status |  |  |  |  |  |  |  |  |
| Cohabitating / Married | 1419/2675 | (53%) | 942/1409 | (67%) | 431/894 | (48%) | 46/372 | (12%) |
| Never married | 1223/2675 | (46%) | 455/1409 | (32%) | 449/894 | (50%) | 319/372 | (86%) |
| Divorced | 26/2675 | (<1%) | 10/1409 | (<1%) | 9/894 | (1%) | 7/372 | (2%) |
| Widowed | 7/2675 | (<1%) | 2/1409 | (<1%) | 5/894 | (<1%) | 0/372 | (0%) |
| Currently attending school | 375/1931 | (19%) | 178/1280 | (14%) | 116/443 | (26%) | 81/208 | (39%) |
| Clinic type |  |  |  |  |  |  |  |  |
| Public | 3158 | (38%) | 2278 | (100%) | 880 | (80%) | 0 | (0%) |
| Private | 5204 | (62%) | 0 | (0%) | 216 | (20%) | 4988 | (100%) |
| Clinic volume |  |  |  |  |  |  |  |  |
| High | 5138 | (61%) | 1831 | (80%) | 880 | (80%) | 2427 | (49%) |
| Low | 3224 | (39%) | 447 | (20%) | 216 | (20%) | 2561 | (51%) |
| In PrEP Cohort | 1178 | (14%) | 513 | (23%) | 309 | (28%) | 356 | (7%) |
| In Cluster randomized trial cohort | 655 | (8%) | 238 | (10%) | 157 | (14%) | 260 | (5%) |
| In Research Cohort | 401 | (5%) | 209 | (9%) | 148 | (14%) | 44 | (<1%) |

**Supplementary table 3: Baseline characteristics of PrEP Cohort by Cohort inclusion**

|  | **Total (n=1178)**  **N (%) or Median (IQR)** | | **CRT Cohort (n=655)**  **N (%) or Median (IQR)** | | **Not CRT Cohort (n=523)**  **N (%) or Median (IQR)** | |
| --- | --- | --- | --- | --- | --- | --- |
| Research technical assistance partner |  |  |  |  |  |  |
| KEMRI Thika | 513 | (44%) | 238 | (36%) | 275 | (53%) |
| KEMRI Kisumu | 309 | (26%) | 157 | (24%) | 152 | (29%) |
| Marie Stopes Kenya | 356 | (30%) | 260 | (40%) | 96 | (18%) |
| Clinic type |  |  |  |  |  |  |
| Public | 741 | (63%) | 383 | (58%) | 358 | (68%) |
| Private | 437 | (37%) | 272 | (42%) | 165 | (32%) |
| Clinic volume |  |  |  |  |  |  |
| High | 814 | (69%) | 500 | (76%) | 314 | (60%) |
| Low | 364 | (31%) | 155 | (24%) | 209 | (40%) |
| Age, Median (IQR) | 23 | (20–26) | 22 | (20–25) | 23 | (21–26) |
| Marital status |  |  |  |  |  |  |
| Cohabitating / Married | 368/979 | (38%) | 190/603 | (32%) | 178/376 | (47%) |
| Never married | 586/979 | (60%) | 394/603 | (65%) | 192/376 | (51%) |
| Divorced | 20/979 | (2%) | 19/603 | (3%) | 1/376 | (<1%) |
| Widowed | 5/979 | (<1%) | 0/603 | (0%) | 5/376 | (1%) |
| Currently attending school | 167/716 | (23%) | 108/466 | (23%) | 59/250 | (24%) |

*Denominator provided if data are missing.*

**Supplementary table 4. Baseline characteristics of CRT Cohort by Research Cohort inclusion**

|  | **Total (n=655)** | | **Research Cohort (n=380)** | | **Not Research Cohort (n=275)** | |
| --- | --- | --- | --- | --- | --- | --- |
|  | n/N | (%) | n/N | (%) | n/N | (%) |
| Research technical assistance partner |  |  |  |  |  |  |
| KEMRI Thika | 238 | (36%) | 201 | (53%) | 37 | (13%) |
| KEMRI Kisumu | 157 | (24%) | 135 | (36%) | 22 | (8%) |
| Marie Stopes Kenya | 260 | (40%) | 44 | (12%) | 216 | (79%) |
| Clinic type |  |  |  |  |  |  |
| Public | 383 | (58%) | 325 | (86%) | 58 | (21%) |
| Private | 272 | (42%) | 55 | (14%) | 217 | (79%) |
| Clinic volume |  |  |  |  |  |  |
| High | 500 | (76%) | 336 | (88%) | 164 | (60%) |
| Low | 155 | (24%) | 44 | (12%) | 111 | (40%) |
| Age, median (IQR) | 22 | (20–25) | 22 | (20–25) | 23 | (20–26) |
| Marital status |  |  |  |  |  |  |
| Cohabitating / Married | 190/603 | (32%) | 146/377 | (39%) | 44/226 | (19%) |
| Never married | 394/603 | (65%) | 217/377 | (58%) | 177/226 | (78%) |
| Divorced | 19/603 | (3%) | 14/377 | (4%) | 5/226 | (2%) |
| Widowed | 0/603 | (0%) | 0/377 | (0%) | 0/226 | (0%) |
| Currently attending school | 108/466 | (23%) | 79/370 | (21%) | 29/96 | (30%) |
| Sex partner living with HIV | 0 | (0%) | 0 | (0%) | 0 | (0%) |
| Sex partner high HIV risk, unknown status | 514 | (78%) | 346 | (91%) | 168 | (61%) |
| Multiple sex partners | 79 | (12%) | 36 | (9%) | 43 | (16%) |
| Ongoing experience with intimate partner violence/gender-based violence | 7 | (1%) | 7 | (2%) | 0 | (0%) |
| Transactional sex | 14 | (2%) | 6 | (2%) | 8 | (3%) |
| STI in the prior 6 months | 37 | (6%) | 30 | (8%) | 7 | (3%) |
| Recurrent use of PEP | 12 | (2%) | 3 | (<1%) | 9 | (3%) |
| Recurrent sex with alcohol/rec drugs | 11 | (2%) | 3 | (<1%) | 8 | (3%) |
| Inconsistent or no condom use | 316 | (48%) | 240 | (63%) | 76 | (28%) |
| Injection drug use with shared needles | 0 | (0%) | 0 | (0%) | 0 | (0%) |
| Signs/symptoms of STI | 2/639 | (<1%) | 2/377 | (<1%) | 0/262 | (0%) |
| Tested negative for HIV | 653 | (>99%) | 380 | (100%) | 273 | (>99%) |
| *Denominator provided if data are missing.* |  |  |  |  |  |  |
